# Supplementary material for: Dehydration Impairs Physical Growth and Cognitive Development in Young Mice
Source: Nutrients. 2020 Feb 29;12(3):670. doi: 10.3390/nu12030670 (PMC7146499; doi:10.3390/nu12030670)
Supplement: Supplementary file 1 [file nutrients-12-00670-s001.pdf]

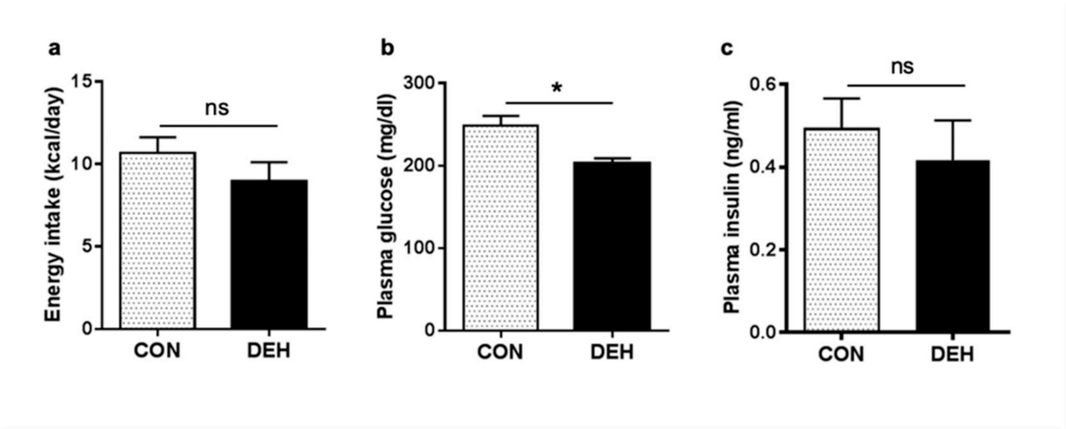

Supplementary Figure 1. Prolonged dehydration induces hypoglycemia. (a)The average daily energy intake during the experimental period was measured in the control (CON) and dehydration (DEH) group. (b, c) Plasma (b) glucose and (c) insulin levels were measured using a dry-chemistry blood analyzer, and via an enzyme-linked immunosorbent assay (ELISA). Data are presented as the mean ± SEM. Statistical significance was evaluated via a Student's t-test: *$p<0.05$ versus CON group. NS, not statistically significant.
